# Thymosin alpha 1 alleviates inflammation and prevents infection in patients with severe acute pancreatitis through immune regulation: a systematic review and meta-analysis

**DOI:** 10.3389/fimmu.2025.1571456

**Published:** 2025-06-17

**Authors:** Yong Tian, Jiaqi Yao, Yihan Ma, Pengcheng Zhang, Xiaofang Zhou, Wenjie Xie, Wenfu Tang

**Affiliations:** ^1^ Department of Integrated Traditional Chinese and Western Medicine, West China Hospital, Sichuan University, Chengdu, Sichuan, China; ^2^ Department of Gastroenterology and Hepatology, Chengdu First People’s Hospital, Chengdu, Sichuan, China; ^3^ Department of General Surgery, The Third Hospital of Mianyang Sichuan Mental Health Center, Mianyang, Sichuan, China; ^4^ West China Center of Excellence for Pancreatitis, West China Hospital, Sichuan University, Chengdu, Sichuan, China

**Keywords:** thymosin alpha 1, inflammation, infection, severe acute pancreatitis, immune regulation, meta-analysis

## Abstract

**Background:**

Immune and inflammatory disorders are part of the complex pathophysiological processes that exacerbate severe acute pancreatitis (SAP) and subsequent infection. Thymosin alpha 1 (Tα1) is an important immunomodulatory agent in clinical practice, but there is a lack evidence to prove its effectiveness in improving the condition of SAP patients. In this study, we aimed to evaluate the efficacy in meta-analysis.

**Methods:**

We systematically searched PubMed, Embase, Web of Science, Cochrane Library and China National Knowledge Infrastructure (CNKI) up to February 1, 2025. Randomized controlled studies comparing the efficacy of Tα1 as intervention measure with non-Tα1 in improving immune regulation for patients with SAP were included. Review Manager 5.3 was used to assess endpoints in the meta-analysis.

**Results:**

Five randomized controlled trials comprising 706 patients with SAP were included. The results indicated that Tα1 could increase the percentages of CD4^+^ cells (MD=4.53, 95%CI [3.02, 6.04], P<0.00001) and improve the CD4^+^/CD8^+^ ratio (MD=0.42, 95%CI [0.26, 0.58], P<0.00001) in SAP patients. There was no statistically significant decrease in CD8^+^ cells. For inflammation, lower-dose Tα1 could significantly reduce C-reactive protein (CRP) levels (mg/L) (MD=-30.12, 95%CI [-35.75, -24.49], P<0.00001), while higher-dose Tα1 showed no statistically significant difference (MD=-3.83, 95%CI [-12.14, 4.49], P=0.37). In terms of infection, the immunomodulatory therapy of Tα1 obviously reduced the overall incidence of extrapancreatic infections in SAP patients (RR=0.56, 95%CI [0.40, 0.78], P=0.0005), especially for blood (RR=0.60, 95%CI [0.38, 0.94], P=0.03) and abdominal (RR=0.38, 95%CI [0.19, 0.78], P<0.0001), while the reduction in lung infections was not statistically significant. Regarding hospital stay (days), Tα1 did not significantly reduce the time spent (MD=-4.22, 95%CI [-11.53, 3.10], P=0.26). However, Tα1 reduced the APACHE II score (MD=-1.52, 95%CI [-2.22, -0.83], P<0.0001).

**Conclusion:**

Tα1 can regulate the balance of immune cells and alleviate immune suppression in SAP patients, including increasing CD4^+^ T cells and CD4^+^/CD8^+^ ratios. Tα1 may exert anti-inflammatory and extrapancreatic infection-preventive effects on SAP patients and improve their condition or prognosis. More researches are needed to validate the results.

**Systematic review registration:**

https://www.crd.york.ac.uk/prospero, identifier CRD42024570517.

## Introduction

Acute pancreatitis (AP) is mainly caused by premature activation of pancreatic enzymes and self-digestion due to factors such as gallstones, hypertriglyceridemia, and alcohol (1–5). The process triggers local pancreatic tissue or systemic inflammatory responses, often manifested as symptoms such as abdominal pain, bloating, nausea, vomiting, and even shock (6). The incidence rate of AP was reported to increase all over the world, with an increase of about 3.07% from 1956 to 2016 (7). About 20% to 30% of the patients are severe acute pancreatitis (SAP), with a mortality rate of over 30% (8, 9). Although the proportion of SAP is lower than that of mild or moderate cases, it has the characteristics of complex disease course, poor prognosis, and high mortality rate.

SAP is often accompanied by systemic inflammatory response syndrome (SIRS), persistent organ failure (POF), and severe complications. SIRS is a significant predictor of poor prognosis in SAP, with the majority of deaths due to multiple organ dysfunction syndrome (MODS). Multi-center international studies have shown that 58% of patients with AP exhibit SIRS, 11% progress to POF, and 2.5% of patients ultimately die from the disease (10, 11). Both early onset or persistent SIRS, and a highest SIRS score of 3 or higher, are independently associated with an increased risk of POF. A retrospective analysis (12) in the United States revealed that the overall prevalence of organ failure in SAP patients was as high as 52%, with those suffering from multiple organ failure experiencing longer hospital stays and a higher mortality risk compared to those without organ failure. Acute necrotizing pancreatitis is characterized by significant necrosis of pancreatic parenchyma and peripancreatic tissue. It may initially manifest as a SIRS similar to SAP, but more often leads to infection. It was reported that approximately 30%-40% of patients with necrotizing pancreatitis were at risk of developing infected pancreatic necrosis, with infected patients facing more than double the mortality risk of those without infection (13, 14). Consequently, curtailing the incidence of SIRS and infectious complications is a crucial strategy for enhancing the prognosis of SAP patients.

Imbalance in the regulation of immune inflammatory response in the body, whether it is pro-inflammatory or anti-inflammatory response dominant, may lead to the deterioration or even death of SAP disease (15). Immunomodulatory therapy is considered an important means in improving the prognosis of SAP patients, such as targeting immune cells and using mesenchymal stem cells for regulation (16). It has been found that decrease in CD4^+^ T lymphocyte levels and CD4^+^/CD8^+^ T lymphocyte ratio indicates a poor prognosis (17). Biological research has demonstrated that thymosin alpha 1 (Tα1) can stimulate the proliferation, differentiation, and maturation of T cells in the thymus (18). This process may elevate CD4^+^ T lymphocyte levels and bolster the CD4^+^/CD8^+^ ratio, thereby enhancing immune function. Previous clinical studies (19–26) have found that Tα1 has a beneficial immunomodulatory effect in patients with a variety of diseases, including malignant tumors, sepsis-induced lung injury, and COVID-19 (**Table 1**). However, there is insufficient evidence to suggest that it has beneficial therapeutic effect in patients with SAP. Therefore, we conducted a meta-analysis of Tα1 for immunomodulatory therapy in SAP patients.

**Table 1:** Potential immunomodulatory effects of thymosin alpha 1 on some diseases.

Study	Disease	Findings
Wei Y.T. et al (19), 2022	Malignant tumor	Tα1 improves the curative effect of chemotherapy by reversing efferocytosis-induced M2 polarization of macrophages via activation of a TLR7/SHIP1 axis.
Yang X. et al (20), 2012	Gastric carcinoma	Tα1 increased the percentage of CD4^+^CD25^+^Foxp3^+^ (suppressive antitumor-specific Tregs), Tregs, IL-1β, TNF-α, and IL-6 in patients with gastric carcinoma.
Zhang Y. et al (21), 2023	Sepsis-induced lung injury	Tα1 inhibits the expressions of TNF-α and IL-6 in sepsis rats and weakens the activity of the Notch signaling pathway, thereby preventing the progression of inflammation and alleviating sepsis-induced lung injury.
Shi Q.X. et al (22), 2020	Traumatic brain injury	Tα1 improves neurological deficits after bTBI in rats due to its inhibition of tau phosphorylation at the Thr205 epitope, increased Treg cells and decreased inflammatory reactions and brain edema.
Giacomini E. et al (23), 2018	Multiple sclerosis	Tα1 treatment enhanced expansion of CD19^+^CD24^+^CD38^hi^ transitional-immature and CD24^low/neg^CD38^hi^ plasmablast-like regulatory B cell subsets, thus inducing anti-inflammatory status and improving multiple sclerosis.
Carraro G. et al (24), 2012	H1N1v influenza	Tα1 enhanced the immunogenicity of the pandemic influenza vaccine used, with good safety and tolerability.
Liu Y. et al (25), 2020	COVID-19	Tα1 reversed T-cell exhaustion (including CD8^+^ T and CD4^+^ T cells) and recovered immune reconstitution through promoting thymus output during severe acute respiratory syndrome-coronavirus 2 infection. Tα1 treatment significantly reduced mortality of severe COVID-19 patients.
Espinar-Buitrago M.S. et al (26), 2023	SARS-Cov2	Tα1 could reduce, through the modulation of dendritic cells, the amount of proinflammatory cytokines produced by T cells. Moreover, Tα1 improve lymphocyte functionality and could become a beneficial therapeutic alternative as an adjuvant in SARS-CoV2 treatment either in the acute phase after infection or reinfection.

(Tα1, thymosin alpha 1; NK, natural killer; IL, interleukin; TNF, tumor necrosis factor; COVID-19, corona virus disease 2019; bTBI, blast induced traumatic brain injury.)

## Materials and methods

### Retrieval strategy

To ensure the comprehensiveness and timeliness of our research, our literature search work covered PubMed, Embase, Web of Science, and Cochrane Library, China National Knowledge Infrastructure (CNKI), from the starting point of each database until February 1, 2025. The following were the English search terms for this study: (‘severe acute pancreatitis’ OR ‘acute pancreatitis’ OR ‘severe pancreatitis’ OR ‘SAP’ OR ‘pancreatic necrosis’ OR ‘pancreatic infection’) AND (‘thymosin alpha 1’ OR ‘thymosin α1’ OR ‘Talpha1’ OR ‘Tα1’ OR ‘TA1’ OR ‘thymus hormones’). The specific retrieval strategy for each database can be found in Supplementary Appendix 1. No language or country restrictions were imposed during the search process. Related publications comparing Tα1 with non-Tα1 treatment (including placebo or standard treatment) to improve inflammation or infection in patients with SAP were considered. Some publications with reliable data from other sources such as grey literature, unpublished studies, or ongoing clinical trials would also be comprehensively considered. In this study, Tα1 and non-Tα1 treatment were used as intervention and control measures, respectively. Changes in percentages of lymphocyte (including CD4^+^, CD8^+^, and CD4^+^/CD8^+^), C-reactive protein (CRP) levels, and number of infections (including blood, lungs, and abdominal cavity) after treatment were primary outcomes. The length of hospital stay and Acute Physiology and Chronic Health Evaluation II (APACHE II) score were secondary outcomes. We performed the meta-analysis based on Preferred Reporting Item for Systematic Reviews and Meta Analyses (PRISMA) statement to ensure the high quality of our work (27, 28). This study has been registered in the International Prospective Systematic Reviews Registry (PROSPERO) with registration number CRD42024570517.

### Inclusion and exclusion criteria

Two researchers reviewed potential and relevant manuscripts that had been published. Studies that met the following selection criteria would be included in the meta-analysis: (1) RCTs evaluating the immunomodulatory therapy of Tα1. (2) The research subjects were SAP patients without gender, age, race, or regional restrictions. (3) The research content included evaluating the efficacy comparison of Tα1 and non-Tα1 in improving inflammation or infection prevention in SAP patients. The exclusion criteria were as follows: (1) Studies that did not meet the inclusion criteria, such as without Tα1 intervention or patients with pancreatitis, would be excluded. (2) Patients with mild acute pancreatitis (MAP) or moderately severe acute pancreatitis (MSAP) would be excluded based on the severity of their condition. In addition, pancreatic cancer patients would also be excluded. (3) Duplicate publications, review articles, editorials, case reports, and animal experiments were excluded. The process of including or excluding published studies was independently completed by two researchers. Any disagreements would be resolved through mutual discussion or consultation with a third author to reach a consensus.

### Data extraction and quality assessment

Thorough examination of the selected studies, two reviewers meticulously extracted the necessary data using a standardized table format. The essential data points included were as follows: the lead author’s name, publication year, sample size, treatment duration, intervention types and dosages, lymphocyte percentages around one week and final percentages (including specifics for CD4^+^, CD8^+^, and the CD4^+^/CD8^+^ ratio), CRP levels around one week and final levels, the count of patients with infections (encompassing blood, lung, and abdominal infections), length of hospital stay, and the APACHE II score. Herein, lymphocyte percentages, CRP levels, and number of infections were examined as the main outcomes. The length of hospital stay and APACHE II score were the additional outcomes. Any ambiguous data that needs to be supplemented or clarified was provided with more details by contacting the corresponding author. For quality assessment of the studies, Jadad scale including the generation of random sequences, randomization concealment, blinding, withdrawal and dropout was used to score the quality of each study. 1–3 points were considered low quality, and 4–7 points were considered high quality.

### Data analysis and publication bias

We conducted statistical analysis on the data using Review Manager version 5.3. We utilized Risk Ratio (RR) for the analysis of dichotomous data and Weighted Mean Difference (WMD) or Standardized Mean Difference (SMD) for continuous data, with a 95% confidence interval (CI) for both. Cochrane’s Q-test and the Inconsistency index (I²) were employed to assess statistical heterogeneity among the included studies. Heterogeneity was considered low if the I² value was less than 50% (I²<50%) and the p-value was greater than 0.1, in which case a fixed-effects model was applied for the pooled analysis. If moderate heterogeneity (75%≥I²≥50%) was detected, a random-effects model was selected. Furthermore, if high heterogeneity (I²>75%) was indicated, we would conduct subgroup analysis or sensitivity analysis to reveal the potential sources of this variability. If clinical and methodological homogeneity was maintained despite statistical heterogeneity, a random-effects model was utilized to offer a more cautious interpretation of intervention effects. All P-values were two-tailed, and a P-value of less than 0.05 was considered to indicate statistical significance. Moreover, we selected CD4^+^ percentages and CRP levels to examine publication bias using Egger’s test of Stata 14.0. And we used Cochrane Risk of Bias (RoB) 2.0 for qualitative bias assessment (29).

## Results

### Literature search and screening

**Figure 1:**
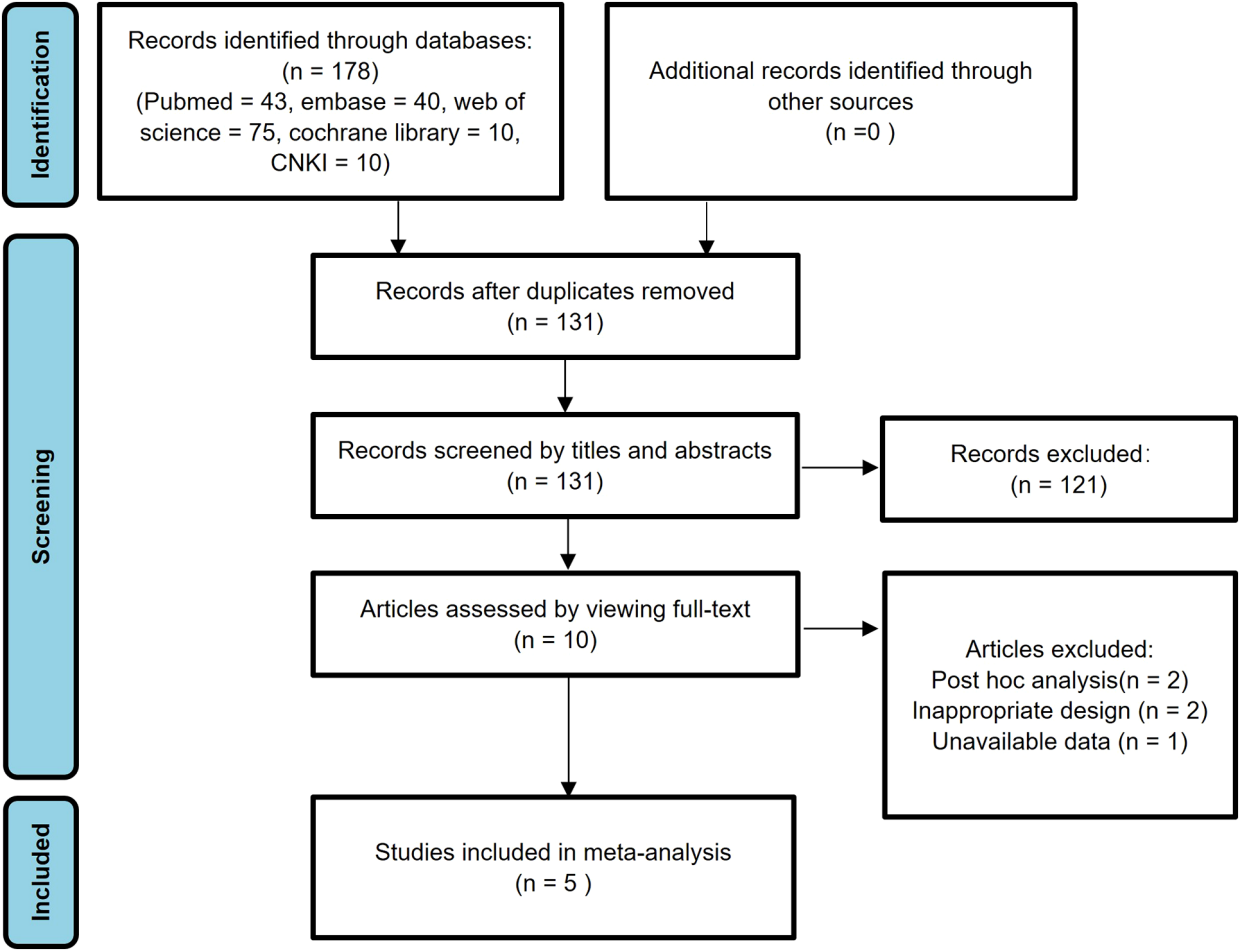
PRISMA flow diagram for study design and literature search.

In the initial search, a total of 178 studies were identified. Subsequently, 47 studies were excluded due to duplicate publications. After reviewing the titles and abstracts, an additional 121 studies were excluded due to irrelevant research content, animal experiments, reviews, comments, and case reports. After carefully examining the full texts of the remaining 10 studies, 2 *post hoc* analysis studies and 2 poorly designed studies were further excluded. Furthermore, one study was excluded due to the unavailability of data. Ultimately, the meta-analysis encompassed 5 eligible published studies (30–34). **Figure 1** provides a visual representation of the research selection process, detailing each stage of study identification, screening, and exclusion.

### Literature characteristics and quality assessment

The analysis comprised 5 studies (30–34), encompassing a total of 706 patients with SAP. All of the five studies were RCTs, with four of them being high-quality articles (30–32, 34) and one assessed as low-quality (33). In addition, two of them were from English databases (30, 31), and three were published in Chinese (32–34). Their Chinese names could be found in **Supplementary Table S1**. These manuscripts were primarily published as full-text articles from 2011 to 2024. In the intervention group, Tα1 was administered via a separate subcutaneous injection as the main measure, whereas the control group received either a placebo or standard treatment alone. All participants in the studies were classified as severe patients. There were no significant differences in the baseline data between the intervention and control groups (including age, gender, partial etiology and lab values), ensuring a fair comparison for the meta-analysis. We extracted the foundational data from the included articles and conducted a thorough quality assessment using Jadad score, as detailed in **Table 2**.

**Table 2:** Characteristics of literatures and quality assessment.

Study	Year	Study design	Included patients	Mean ages	Regimens	Patients of group	Medication time	Jadad score
Ke L. et al (30)	2022	RCT	508	T: 44.3 ± 13.2	Subcutaneous injection of Tα1 1.6 mg every 12 h for the first 7 days and 1.6 mg once a day for the following 7 days	254	≤14 days	7
				C: 45.4 ± 13.4	Placebo	254	≤14 days	
Wang X. et al (31)	2011	RCT	24	T: 42.0 ± 8.0	Subcutaneous injection of Tα1 3.2 mg twice a day for 7 days.	12	7 days	5
				C: 50.0 ± 11.0	Placebo	12	7 days	
Yuan J. et al (32)	2021	RCT	40	T: 47.30 ± 8.62	Subcutaneous injection of Tα1 3.2 mg once a day plus standard treatment	20	14 days	4
				C: 43.55 ± 9.60	Standard treatment	20	14 days	
Lv Z. et al (33)	2011	RCT	50	T: NA	Subcutaneous injection of Tα1 1.6 mg once a day plus standard treatment	25	7 days	3
				C: NA	Standard treatment with Sandostatin intravenous drip once a day	25	7 days	
Huang Y. et al (34)	2024	RCT	84	T: 52.42 ± 12.15	Subcutaneous injection of Tα1 1.6mg once a day in the first week, every other day from the second week plus standard treatment	42	14 days	4
				C: 51.86 ± 11.52	Standard treatment	42	14 days	

(RCT, randomized controlled trial; T, trial group; C, control group; NA, no availability; Tα1, thymosin alpha 1.)

### Lymphocyte percentages

**Figure 2:**
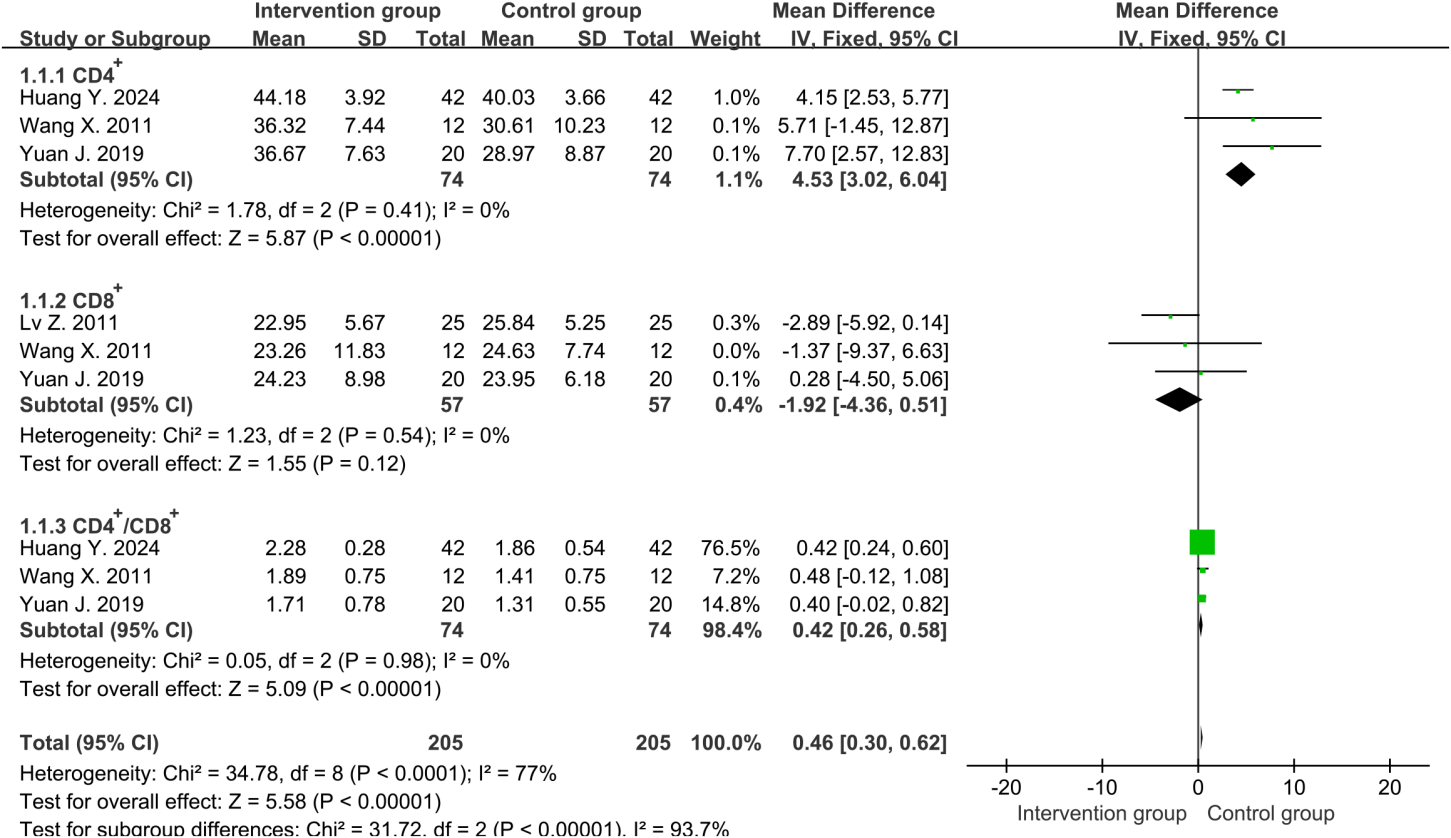
Forest plot of lymphocyte percentages around one week (including CD4^+^, CD8^+^ and CD4^+^/CD8^+^ ratio).

Four studies (31–34) reported the percentages of CD4^+^ and three studies (31–33) reported the percentages of CD8^+^ around one week. The average percentages of CD4^+^ in the intervention group and the control group were approximately 46.9% and 36.7%, while the percentages for CD8^+^ were 23.5% and 25.0%, respectively. High heterogeneity was determined using Cochrane’s Q-test for two groups of CD4^+^ cells (degrees of freedom [df]=3, I^2^ = 98%, P<0.00001), while CD8^+^ cells showed low heterogeneity (df=2, I^2^ = 0%, P=0.54). However, when sensitivity analysis was conducted on studies reporting CD4^+^ cells, heterogeneity was significantly reduced after excluding one study (33) (df=2, I^2^ = 0%, P=0.41). Subsequently, we chose fixed-effects analysis for both CD4^+^ and CD8^+^ cells. The results showed that compared with the control group, the percentages of CD4^+^ in the treatment group increased significantly and had statistical differences (MD=4.53, 95%CI [3.02, 6.04], P<0.00001) (**Figure 2**), while CD8^+^ cells decreased slightly and did not reach statistical differences (MD=-1.92, 95%CI [-4.36, 0.51], P=0.12) (**Figure 2**). Even if we excluded the same study as CD4^+^ cells again, there was still no statistically significant difference in the trend of CD8^+^ results (MD=-0.15, 95%CI [-4.26, 3.95], P=0.94). Therefore, we chose to keep it in our analysis.

Four studies (31–34) reported the ratio of CD4^+^ to CD8^+^ around one week. High heterogeneity was discovered among them (df=3, I^2^ = 90%, P<0.00001). However, heterogeneity was significantly reduced after excluding one study (33) (df=2, I^2^ = 0%, P=0.98). The results in fixed-effects showed that the CD4^+^/CD8^+^ ratio in the intervention group was significantly higher than that in the control group (MD=0.42, 95%CI [0.26, 0.58], P<0.00001) (**Figure 2**).

Similarly, we analyzed the final lymphocyte percentages (including CD4^+^, CD8^+^ and CD4^+^/CD8^+^ ratio). The analysis conclusions are basically similar to the results around one week (**Supplementary Figure S1**).

### CRP levels

**Figure 3:**
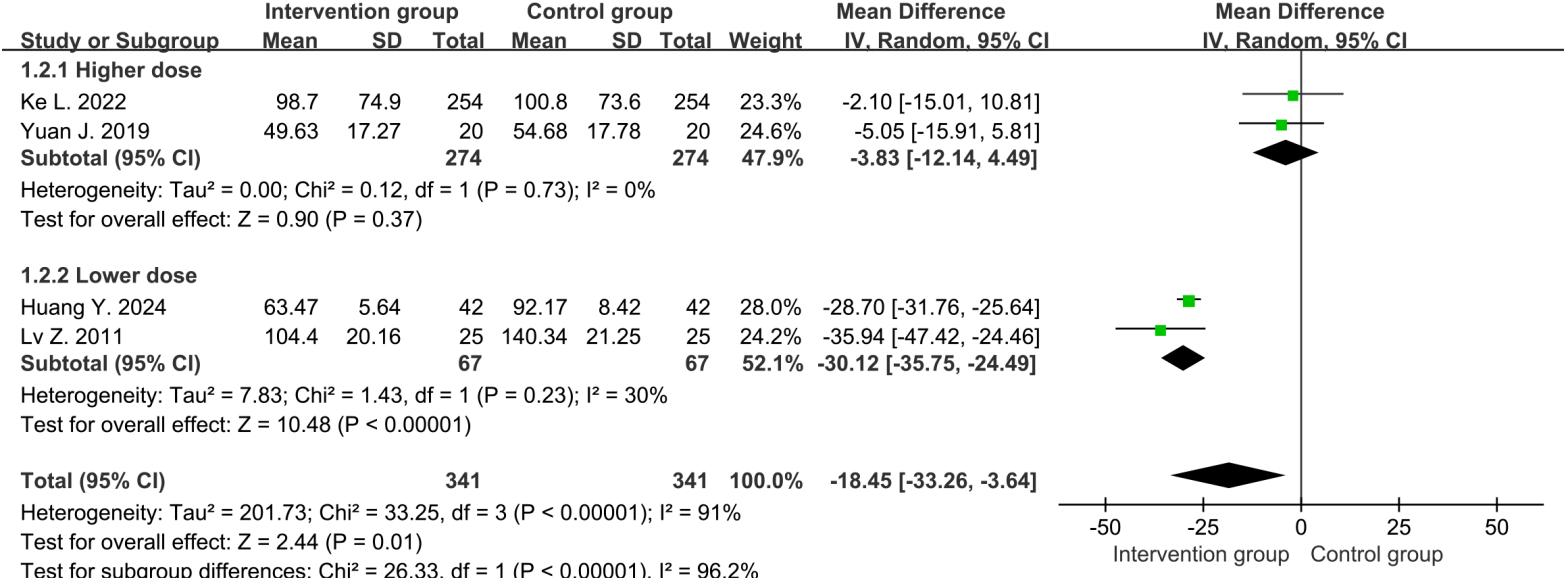
Forest plot of C-reactive protein levels around one week (including subgroup analysis of higher and lower doses).

Four studies (30, 32–34) reported the CRP levels (mg/L) around one week, with an average of 91.9mg/L and 100.0mg/L in the intervention and control groups, respectively. There was high heterogeneity between two groups (df=3, I^2^ = 91%, P<0.00001). Through sensitivity analysis, heterogeneity was not significantly reduced. We divided the study into higher-dose (3.2 mg per day) and lower-dose (1.6 mg per day) subgroups, with significant reduction in heterogeneity. Therefore, it was possible that the heterogeneity source was caused by drug dosage. The results in random-effects showed that the overall levels of CRP in the intervention group were lower than those in the control group (MD=-18.45, 95%CI [-33.26, -3.64], P=0.01) (**Figure 3**). Subgroup analysis showed that the lower-dose group performed more significantly (MD=-30.12, 95%CI [-35.75, -24.49], P<0.00001), while the higher-dose group showed no statistical difference (MD=-3.83, 95%CI [-12.14, 4.49], P=0.37).

In addition, we analyzed the final CRP levels (mg/L). The final conclusions of the overall levels of CRP and subgroup analysis are consistent with those results around one week (**Supplementary Figure S2**).

### Patients with infections

**Figure 4:**
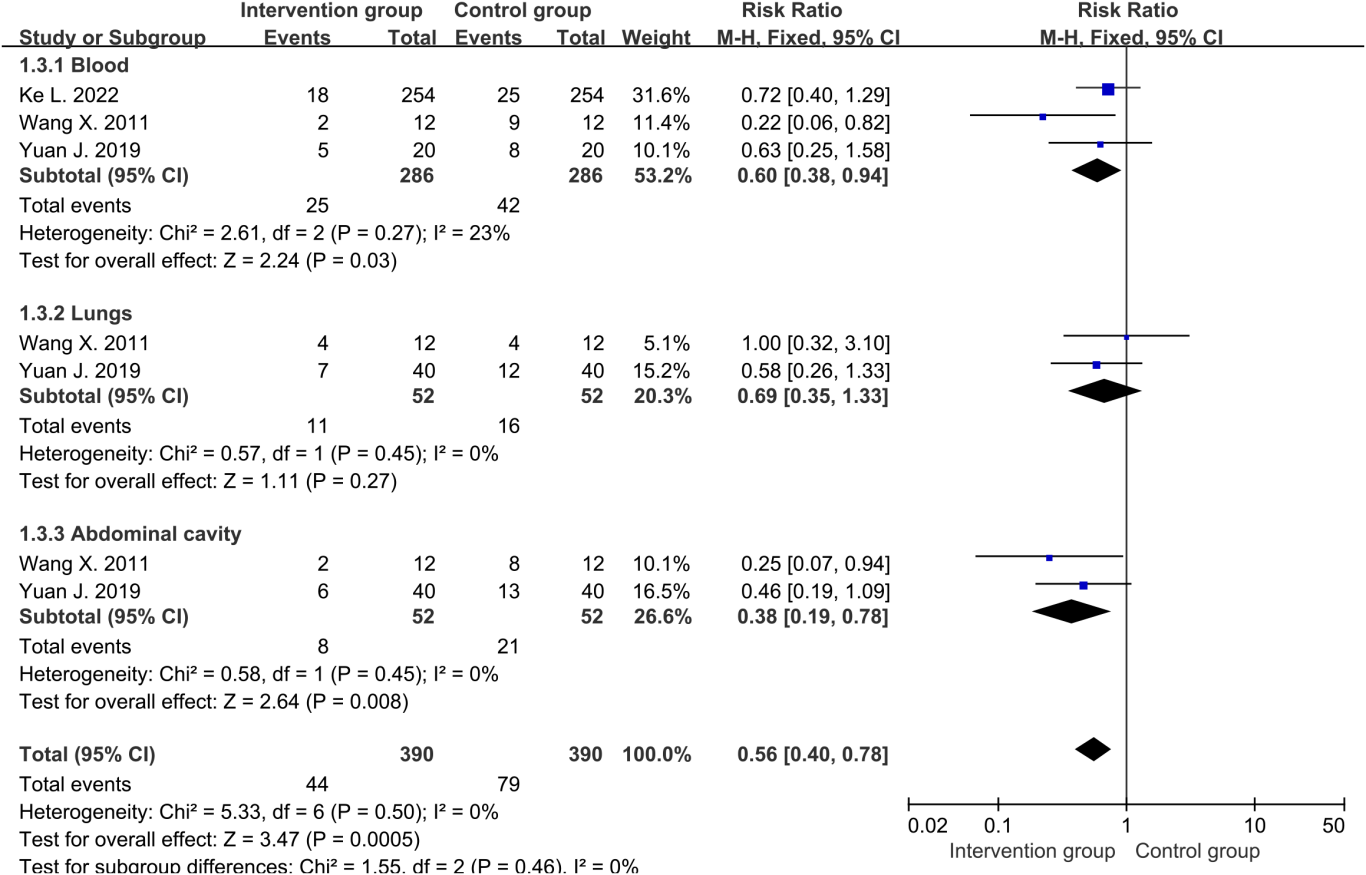
Forest plot of patients with infections.

There were three studies (30–32) reporting the final number of blood infections, and two studies (31, 32) reporting the number of lung and abdominal infections. The average infection rate of the intervention group was about 14.3%, while that of the control group was about 25.9%. Cochrane’s Q-test revealed low heterogeneity among studies involving blood, lungs, and abdominal cavity. Therefore, fixed-effects were used to analyze them. The results showed that the overall infection rate of the intervention group was significantly lower than that of the control group (RR=0.56, 95%CI [0.40, 0.78], P=0.0005) (**Figure 4**), with less infections of blood (RR=0.60, 95%CI [0.38, 0.94], P=0.03) and abdominal (RR=0.38, 95%CI [0.19, 0.78], P=0.008) being the most significant. There was a relatively lower trend of lung infection in intervention group, but it had not reached statistical significance (RR=0.69, 95%CI [0.35, 1.33], P=0.27).

### Length of hospital stay

**Figure 5:**
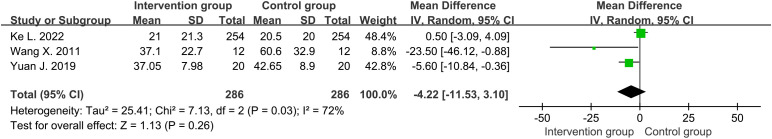
Forest plot of hospital stay.

Three studies (30–32) reported the overall length of hospital stay (days) for SAP patients. The average length of hospital stay in the intervention group was about 22.8 days, while the control group was about 23.7 days. Moderate heterogeneity was detected among the included studies (df=2, I^2^ = 72%, P=0.03). No significant difference was found in the overall length of hospital stay between intervention group and control group through random-effects (MD=-4.22, 95%CI [-11.53, 3.10], P=0.26) (**Figure 5**)

### APACHE II score

**Figure 6:**
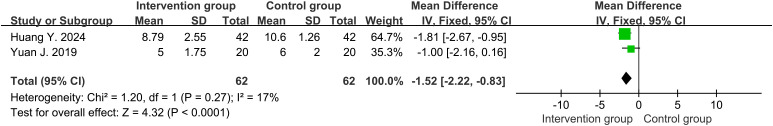
Forest plot of APACHE II score.

Three studies (32–34) reported the APACHE II score. High heterogeneity was discovered among them (df=2, I^2^ = 93%, P<0.00001). The results in random-effects showed that the intervention group had lower APACHE II score than the control group (MD=-3.37, 95%CI [-6.24, -0.49], P=0.02) (**Supplementary Figure S3**). Heterogeneity significantly decreased when a study (33) was excluded (df=1, I^2^ = 17%, P=0.27). Its overall treatment time (7 days) was shorter than the other two studies (10 and 14 days), which might be a source of heterogeneity. The results using a fixed-effects model still led to the same conclusion (MD=-1.52, 95%CI [-2.22, -0.83], P<0.0001) (**Figure 6**).

### Publication bias test

Due to the limited number of studies included, we chose percentages of CD4^+^ and CRP levels to evaluate publication bias. The P-values for percentages of CD4^+^ and CRP levels using Egger test were 0.385 and 0.195 (P>0.05), respectively, indicating that there was no significant publication bias. In addition, partial subgroup analysis (including lymphocyte ratio, inflammation, and infection) based on language was used to test for bias. Meaningful merging results (including two or more) were consistent with the trend of the original results mentioned above (**Supplementary Figures S4**-**S6**). According to Cochrane RoB 2.0 assessment, it was found that the publication bias in English articles was low-risk (**Supplementary Tables S2**, **S3** and **S7**). Some aspects of the Chinese article had ‘some concerns’, but none of them had reached high-risk bias (**Supplementary Tables S4**-**S7**).

## Discussion

Imbalance of immune regulation in the body is an important reason for the progression of pancreatitis to severe or even death in SAP patients. Excessive activation of local inflammatory cells and mediators can lead to increased capillary permeability, exacerbating the inflammatory response and transforming it into SIRS. Persistent SIRS leads to circulatory, respiratory, or renal failure, resulting in MODS and significantly increased mortality rates (35). Compensatory anti-inflammatory response syndrome (CARS), as a negative feedback regulation, can help the body suppress excessive inflammatory reactions (36). However, excessive CARS effect may lead to a decrease in the expression of human leukocyte antigen-DR (HLA-DR), resulting in immune suppression and significantly increasing the risk of infection in the body (37–39). In addition, as an important component of the immune system, the lymphocyte ratio in SAP patients is significantly reduced (40, 41). T cell subpopulation analysis showed that multiple cell lines were inhibited in AP, including cytotoxic CD8^+^ T cells, natural killer (NK) cells, and CD4^+^ T cell counts (42, 43). In severe cases, CD4^+^ T cell counts have been reported to decrease more significantly than CD8^+^ cells, and lead to a decrease in CD4^+^/CD8^+^ ratio (17, 42, 44). Some subsets and functions of CD4^+^ T cells differentiation can be viewed in **Supplementary Figure S7**. The reduction of some differentiation types may further increase the risk of infection and death for SAP patients (45). Therefore, it is necessary to improve the prognosis of SAP patients through immunomodulatory therapy.

At present, the main goal of immunomodulatory therapy is to regulate the maturation, apoptosis, and differentiation of immune cells through immune stimulation, restoring the balance of immune cell quantity and function (46). This method may also be used in combination with anti-inflammatory drugs targeting certain cytokines (including NF-κB, TNF-α, interleukins, and platelet activating factors) for multi strategy treatment (47, 48). Immune stimulation methods such as the use of granulocyte macrophage colony-stimulating factor (GM-CSF) and interferon (IFN)-γ have been reported to increase the expression level of HLA-DR on monocytes or restore the balance between T helper cell 1 (Th1) and Th2 (49, 50). But clinical studies on these findings are still scarce. Interestingly, Tα1 can restore serum CD4^+^ T cell levels and CD4^+^/CD8^+^ ratio, and has also been increasingly used in clinical studies for immune regulation therapy of SAP in recent years (30–34, 51, 52). However, there is currently insufficient evidence to prove its efficacy, and this meta-analysis is needed to explore the immunomodulatory therapeutic effect of Tα1 on SAP patients.

Our research findings suggest that Tα1 may improve immune regulation in SAP patients. Tα1 is a peptide naturally present in the thymus, and it has long been believed to alter, enhance, and restore immune function. Tα1 can serve as an enhancer for immune function decline caused by a decrease in T cell related components. Tα1 interacts with Toll-like receptors (TLRs) and activates dendritic cells and precursor T cells, increasing the number of T helper cells and transferring to Th1 class, thereby increasing the expression of cytokines such as IL-2, IFN-α, and the activity of NK cells (53). Animal experimental studies (52, 54) have shown that Tα1 can alleviate pancreatitis by balancing CD3^+^/CD4^+^/CD8^+^ T cells and reducing cytokine release, reducing cell damage, thereby relieving the severity of the pancreas and improving the survival rate of SAP mice. The condition of AP is closely related to the level of CD4^+^ T lymphocytes, and its possible mechanism is that IL-22 can protect mice from AP invasion, while CD4^+^ T lymphocytes are the main source of IL-22 in pancreatic tissue (55, 56). Our analysis results indicated that the use of Tα1 immunomodulatory therapy significantly increased CD4^+^ T cells, CD4^+^/CD8^+^ ratio around one week, and slightly decreased CD8^+^ T cells levels in SAP patients. The conclusion remained consistent in the final percentages. This suggested that Tα1 might tend to improve the number of CD4^+^ T cells in the peripheral blood of SAP patients, thereby regulating immune balance and preventing immune suppression.

CRP levels increase during plasma inflammation and are a commonly used biomarker for assessing the degree of inflammation in the body. In patients with SAP, a strong negative correlation has been found between CRP levels and the proportion of T helper cells (57). Tα1 possesses the capacity to prevent pro-inflammatory cytokine storms and potential autoimmune events. This is due to its ability to activate indoleamine-2,3-dioxygenase in plasma cell like dendritic cells, leading to the production of IL-10 and an increase in regulatory T cells, and ultimately inhibiting excessive cytokine production (58–60). In addition, it may reduce M1 activation of macrophages and lower the levels of pro-inflammatory cytokines such as TNF - α, IL-1 β and IL-6 (61). In a word, these processes allow for a balanced control of inflammation and tolerance. Our study found that lower doses (1.6 mg per day) of Tα1 significantly reduced CRP levels in SAP patients, while there was no significant difference between the two groups at higher doses (3.2 mg per day). Interestingly, the analysis conclusion of final CRP levels remained consistent with it around one week. It is unknown whether high-dose Tα1 affects the differentiation trend of CD4^+^ T cell subsets in SAP patients and affects therapeutic efficacy. But it can be speculated that low-dose Tα1 may be more used for immune regulation and maintaining immune homeostasis, reducing autoimmune reactions and inflammation in SAP patients. However, it requires more research to confirm.

Infectious pancreatic necrosis (IPN), as a local infection of the pancreas, is mainly caused by secondary infection of pancreatic necrotic tissue. For the infectious necrosis, in addition to using antibiotics, invasive interventions such as percutaneous puncture drainage, endoscopic drainage, or surgical debridement can be used to remove necrotic tissue and infected lesions (62). In the articles we included, a study predicting IPN had the highest number of patients (30). Although patients using Tα1 showed a trend toward lower incidence of IPN compared to those using a placebo during hospitalization (15.7% vs 18.1%) and within 90 days after randomization (22.4% vs 25.6%), there was a lack of statistical difference. And it did not perform well in some invasive interventions. Ke L. et al. (30) proposed that future trials need to determine the selection of the best patient, most effective dose, and duration of Tα1 treatment. These factors may have some impact on the results.

Extrapancreatic infection (EPI) is a common clinical complication in AP patients during hospitalization, referring to infections of other organs except pancreas, including blood, respiratory tract, abdominal cavity, and urinary tract. A meta-analysis (63) of 19 studies involving 1741 patients showed that the incidence of complications from EPI was 32% (95% CI 23-41%), with the most common being respiratory infections (9.2%) and bacteremia (8.4%). Multiple studies (64–66) have found that prophylactic use of antibiotics is common in SAP, but routine early prophylactic antibiotic use does not have significant clinical benefits for SAP patients. The guidelines of the American gastroenterological association institute and European Society of Gastrointestinal Endoscopy (ESGE) in 2018 suggested that prophylactic use of antibiotics was not recommended for patients predicted to have severe or necrotizing pancreatitis (67, 68). However, Tα1, as an immunomodulator, has been widely used and tested in a wide range of clinical applications, including viral, fungal and bacterial infectious diseases (53). Our results found that the overall incidence rate of EPI in SAP patients after Tα1 immunomodulation treatment was about 14.3%, significantly lower than that in the control group (about 25.9%). Specifically, there was a significant difference in preventing blood and abdominal infections, while the effect was slightly lower in preventing pulmonary infections. In addition, based on the results of final lymphocyte percentages (including CD4^+^ T cells, CD4^+^/CD8^+^ratio), patients in the Tα1 group appeared to exhibit less pronounced immunosuppression. Tα1 may reduce the exhaustion of T cells in SAP patients and maintain the number and function of effector T cells, thus playing a sustained role in preventing or eliminating infections. In summary, Tα1 has a certain effect on preventing EPI in SAP patients.

The length of hospital stay in the studies we included was generally between 3 and 4 weeks. The duration is significantly longer than mild to moderate patients (69). Our results indicated that Tα1 had a trend of reducing hospitalization time for SAP patients. However, it is not sufficient to achieve statistical significance. The average length of hospital stay for SAP patients is influenced by multiple factors. A retrospective study found that organ dysfunction at presentation or during admission, concurrent infections, need for enteral tube placement and in-hospital interventions were associated with increased length of hospital stay for acute necrotizing pancreatitis (70). Some studies showed that early enteral nutrition, good control of blood glucose levels and the use of Chinese herbal medicine were associated with decreased length of hospital stay (71–75). However, most of the patients included were severe acute necrotizing pancreatitis, and the study conducted by Ke L. et al. (30) found that there was no statistical difference in the incidence of IPN and some invasive interventions. Perhaps the main impact on hospitalization time may be other factors or intervention measures, and the effect of Tα1 in this regard appears to be relatively weak.

The severity of SAP patients’ condition upon admission needs to be quickly assessed through some scoring criteria. At present, there are still different opinions on the advantages and disadvantages of different scoring systems, including APACHE II score, Bedside Index of Severity in Acute Pancreatitis (BISAP), Ranson’s score and Modified Computed Tomography Severity Index (MCTSI) (76–78). The APACHE II score was mainly mentioned in the studies we included. It scores based on the patient’s physiological parameters, age, and chronic health status. It helps to quickly assess the severity of SAP patients’ conditions and has some value in guiding treatment and prognosis. It is known as the ‘gold standard’ for predicting severely ill patients in individual intensive care units worldwide (79). According to our analysis results, the APACHE II score of the treatment group was relatively lower than that of the control group. We believe that using Tα1 for immune regulation may improve the condition and prognosis of SAP patients.

Regarding the adverse events of Tα1, there is insufficient data to report it in the included study. However, it has been reported that Tα1 has good tolerability in a wide population, including elderly patients, children, and immunocompromised patients, and no any significant adverse events in patients with organ dysfunction (24, 80). Further research is required to substantiate the safety of its use in patients with SAP.

In this study, we chose to analyze some outcomes around one week in addition to the final outcomes. This reduces the impact of inconsistent medication time on the final results, proving the reliability of the conclusion. Although our study has found some beneficial effects of Tα1 on SAP patients as the first meta-analysis, there are still some shortcomings in the study. First, we have included relatively few studies, and more studies are needed to confirm the results. Secondly, due to limited data included in the study, we are unable to comprehensively analyze the efficacy and safety of Tα1. If possible, more outcome measures (such as clinical symptoms, mortality rate, adverse events) could be added in the future. Thirdly, the dosage of Tα1 used in the included studies is not completely consistent, which may lead to high heterogeneity in some results and affect stability. Finally, one study (33) is of low quality and has been assessed as ‘some concerns’ about publication bias according to Jadad scale and Cochrane RoB 2.0. This is also the reason why we excluded it in some result analysis. More high-quality research is needed to validate the immunomodulatory effect of Tα1 on SAP.

## Conclusion

Our findings suggest that Tα1 can regulate the balance between immune cells in SAP patients, including increasing CD4^+^ T cells and CD4^+^/CD8^+^ ratios. Furthermore, Tα1 may exert anti-inflammatory and EPI-preventive effects on SAP patients, and ultimately improve their condition or prognosis. However, more research is needed to validate these results.

## Data Availability

The original contributions presented in the study are included in the article/**Supplementary Material**. Further inquiries can be directed to the corresponding author.
