# Supplementary material for: Thymosin alpha 1 alleviates inflammation and prevents infection in patients with severe acute pancreatitis through immune regulation: a systematic review and meta-analysis
Source: Front Immunol. 2025 Jun 17;16:1571456. doi: 10.3389/fimmu.2025.1571456 (PMC12208829; doi:10.3389/fimmu.2025.1571456)

Supplementary material contents

Supplementary Appendix S1. Search strategies for English and Chinese databases 1

Supplementary Table S1. English comparison table of included Chinese studies in China National Knowledge Infrastructure. 2

Supplementary Figure S1. Forest plot of final lymphocyte percentages (including $CD4^+$, $CD8^+$ and $CD4^+/CD8^+$ ratio) 3

Supplementary Figure S2. Forest plot of final C-reactive protein levels (including subgroup analysis of higher and lower doses) 3

Supplementary Figure S3. Forest plot of APACHE II Score with random-effects........................ 3

Supplementary Figure S4. Subgroup analysis of $CD4^+/CD8^+$ ratio based on language................ 4

Supplementary Figure S5. Subgroup analysis of C-reactive protein levels based on language..... 4

Supplementary Figure S6. Subgroup analysis of positive blood culture based on language 4

Supplementary Table S2. Risk assessment of bias for Ke L. et al.'s study using Cochrane RoB 2.0. 5-7

Supplementary Table S3. Risk assessment of bias for Wang X. et al.'s study using Cochrane RoB 2.0. 8-10

Supplementary Table S4. Risk assessment of bias for Yuan J. et al.'s study using Cochrane RoB 2.0. 11-13

Supplementary Table S5. Risk assessment of bias for Lv Z. et al.'s study using Cochrane RoB 2.0. 14-16

Supplementary Table S6. Risk assessment of bias for Huang Y. et al.'s study using Cochrane RoB 2.0. 17-19

Supplementary Table S7. Overall bias for all studies using Cochrane RoB 2.0. 20

Supplementary Figure S7. Diagram for main subsets and partial functions of CD4+ T cells differentiation.. 21

Supplementary Appendix S1. Search strategies for English and Chinese databases

Overall Strategy for English Database：

#1 ('severe acute pancreatitis' OR 'acute pancreatitis' OR 'severe pancreatitis' OR 'SAP' OR 'pancreatic necrosis' OR 'pancreatic infection')

#2 ('thymosin alpha 1' OR 'thymosin α1' OR 'Talpha1' OR ' Tα1' OR ' TA1'OR 'thymus hormones')

#3 #1 AND #2

Overall Strategy for Chinese Database:

#1（重症急性胰腺炎 OR 重症胰腺炎 OR 胰腺炎 OR 胰腺坏死 OR 胰腺感染）

#2（胸腺肽α1 OR 胸腺肽）

#3 #1 AND #2

1.Pubmed (N=43)

((((((severe acute pancreatitis) OR (acute pancreatitis)) OR (severe pancreatitis)) OR (SAP)) OR (pancreatic necrosis)) OR (pancreatic infection)) AND ((((((thymosin alpha 1) OR (thymosin α1)) OR (Talpha1)) OR (Tα1)) OR (TA1)) OR (thymus hormones))

2.Embase (N=40)

('severe acute pancreatitis'/exp OR 'severe acute pancreatitis' OR 'acute pancreatitis'/exp OR 'acute pancreatitis' OR 'severe pancreatitis' OR 'sap'/exp OR 'sap' OR 'pancreatic necrosis'/exp OR 'pancreatic necrosis' OR 'pancreatic infection'/exp OR 'pancreatic infection') AND ('thymosin alpha 1'/exp OR 'thymosin alpha 1' OR 'thymosin α1' OR 'talpha1' OR 'tα1' OR 'ta1' OR 'thymus hormones'/exp OR 'thymus hormones')

3.Cochrane library (N=10)

((((((severe acute pancreatitis) OR (acute pancreatitis)) OR (severe pancreatitis)) OR (SAP)) OR (pancreatic necrosis)) OR (pancreatic infection)) AND ((((((thymosin alpha 1) OR (thymosin α1)) OR (Talpha1)) OR (Tα1)) OR (TA1)) OR (thymus hormones))

4.Web of science (N=75)

(severe acute pancreatitis OR acute pancreatitis OR severe pancreatitis OR SAP OR pancreatic necrosis OR pancreatic infection) AND (thymosin alpha 1 OR thymosin α1 OR Talpha1 OR Tα1 OR TA1 OR thymus hormones)

5.China National Knowledge Infrastructure (CNKI) (N=10)

（“重症急性胰腺炎” OR “重症胰腺炎” OR “胰腺炎” OR “胰腺坏死” OR “胰腺感染”）AND（“胸腺肽α1” OR “胸腺肽”）

Supplementary Table S1. English comparison table of included Chinese studies in China National Knowledge Infrastructure (CNKI: https://www.cnki.net/)

Reference Number	Comparison between Chinese and English
[32]	Yuan J. Clinical study on the changes of immune function and the role of thymosin α1 in acute pancreatitis. Southwest Medical University (2019).<br>袁景. 急性胰腺炎免疫功能变化及胸腺肽 α1 的作用临床研究. 西南医科大学 (2019).
[33]	Lv Z, Li K, Wang M, et al. Study on the efficacy of thymosin α1 in treating severe acute pancreatitis. J Bethune Military Med Coll. (2011) 9:252–4.<br>吕增发, 李克峰, 王萌, 刘青光. 胸腺肽-α1 治疗急性重症胰腺炎的疗效观察. 白求恩军医学院学报, 2011,9:252-4.
[34]	Huang Y, Ding J, Fan H, et al. The efficacy of Thymosin Alpha 1 in treating severe acute pancreatitis and its impact on patients' cellular immune status and inflammatory mediators. Modern Med J China. (2024) 26:47–50.<br>黄钰萍, 丁江涛, 范辉, 王亚民, 王小红. 胸腺肽 α1 治疗重症急性胰腺炎的疗效及对患者细胞免疫状态、炎症介质的影响. 中国现代医药杂志, 2024,26:47-50.

Supplementary Figure S1. Forest plot of final lymphocyte percentages (including $CD4^+$, $CD4^+$ and $CD4^+/CD8^+$ ratio)

Study or Subgroup	Intervention group Mean	SD	Total	Control group Mean	SD	Total	Weight	Mean Difference IV, Fixed, 95% CI
**1.1.1 CD4$^+$**								
Huang Y. 2024	53.86	4.78	42	44.92	4.1	42	0.8%	8.94 [7.04, 10.84]
Wang X. 2011	36.88	7.92	12	31.03	9.87	12	0.1%	5.85 [-1.31, 13.01]
Yuan J. 2019	38.77	8.5	20	30.86	5.15	20	0.1%	7.91 [3.55, 12.27]
**Subtotal (95% CI)**			**74**			**74**	**1.0%**	**8.61 [6.92, 10.31]**
Heterogeneity: Chi² = 0.79, df = 2 (P = 0.68); I² = 0%								
Test for overall effect: Z = 9.95 (P < 0.00001)								
**1.1.2 CD8$^+$**								
Lv Z. 2011	22.95	5.67	25	25.84	5.25	25	0.3%	-2.89 [-5.92, 0.14]
Wang X. 2011	22.19	8.54	12	23.4	5.7	12	0.1%	-1.21 [-7.02, 4.60]
Yuan J. 2019	22.16	5.77	20	23.62	4.35	20	0.3%	-1.46 [-4.63, 1.71]
**Subtotal (95% CI)**			**57**			**57**	**0.7%**	**-2.08 [-4.13, -0.03]**
Heterogeneity: Chi² = 0.51, df = 2 (P = 0.78); I² = 0%								
Test for overall effect: Z = 1.99 (P = 0.05)								
**1.1.3 CD4$^+$/CD8$^+$**								
Huang Y. 2024	2.95	0.36	42	1.99	0.58	42	66.4%	0.96 [0.75, 1.17]
Wang X. 2011	1.72	0.55	12	1.35	0.65	12	12.2%	0.37 [-0.11, 0.85]
Yuan J. 2019	1.88	0.71	20	1.37	0.49	20	19.8%	0.51 [0.13, 0.89]
**Subtotal (95% CI)**			**74**			**74**	**98.3%**	**0.80 [0.63, 0.97]**
Heterogeneity: Chi² = 7.63, df = 2 (P = 0.02); I² = 74%								
Test for overall effect: Z = 9.20 (P < 0.00001)								
**Total (95% CI)**			**205**			**205**	**100.0%**	**0.85 [0.69, 1.02]**
Heterogeneity: Chi² = 97.67, df = 8 (P < 0.00001); I² = 92%								
Test for overall effect: Z = 9.95 (P < 0.00001)								
Test for subgroup differences: Chi² = 88.75, df = 2 (P < 0.00001), I² = 97.7%								

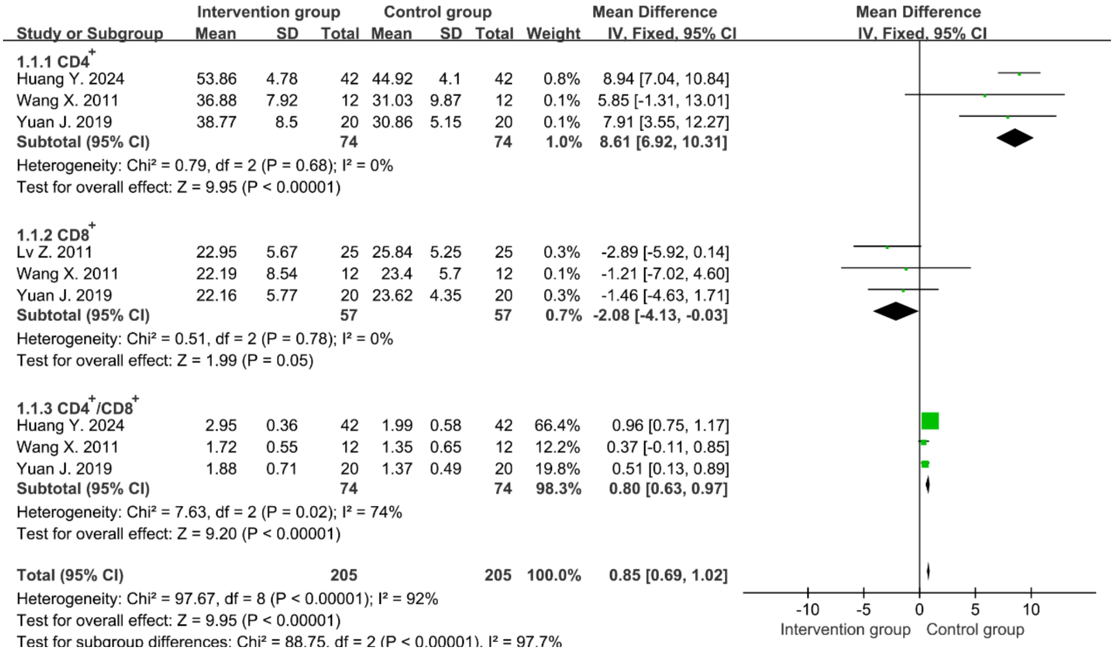

Supplementary Figure S2. Forest plot of final C-reactive protein levels (including subgroup analysis of higher and lower doses)

Study or Subgroup	Intervention group Mean	SD	Total	Control group Mean	SD	Total	Weight	Mean Difference IV, Random, 95% CI
**1.2.1 Higher dose**								
Ke L. 2022	53.6	54.2	254	54.5	54.3	254	24.0%	-0.90 [-10.34, 8.54]
Yuan J. 2019	31.62	8.23	20	43.1	12.1	20	25.9%	-11.48 [-17.89, -5.07]
**Subtotal (95% CI)**			**274**			**274**	**49.8%**	**-6.78 [-17.08, 3.52]**
Heterogeneity: Tau² = 39.03; Chi² = 3.30, df = 1 (P = 0.07); I² = 70%								
Test for overall effect: Z = 1.29 (P = 0.20)								
**1.2.2 Lower dose**								
Huang Y. 2024	18.83	1.67	42	45.21	4.13	42	27.7%	-26.38 [-27.73, -25.03]
Lv Z. 2011	104.4	20.16	25	140.34	21.25	25	22.5%	-35.94 [-47.42, -24.46]
**Subtotal (95% CI)**			**67**			**67**	**50.2%**	**-29.39 [-38.09, -20.69]**
Heterogeneity: Tau² = 28.30; Chi² = 2.63, df = 1 (P = 0.11); I² = 62%								
Test for overall effect: Z = 6.62 (P < 0.00001)								
**Total (95% CI)**			**341**			**341**	**100.0%**	**-18.57 [-31.09, -6.04]**
Heterogeneity: Tau² = 147.20; Chi² = 49.27, df = 3 (P < 0.00001); I² = 94%								
Test for overall effect: Z = 2.91 (P = 0.004)								
Test for subgroup differences: Chi² = 10.80, df = 1 (P = 0.001), I² = 90.7%								

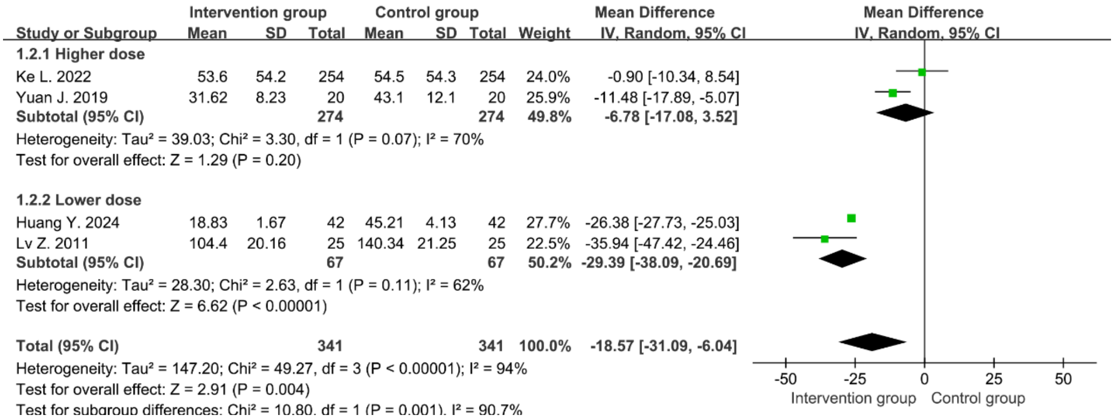

Supplementary Figure S3. Forest plot of APACHE II Score with random-effects

Study or Subgroup	Intervention group Mean	SD	Total	Control group Mean	SD	Total	Weight	Mean Difference IV, Random, 95% CI
Huang Y. 2024	8.79	2.55	42	10.6	1.26	42	35.5%	-1.81 [-2.67, -0.95]
Lv Z. 2011	8.55	4.62	25	16.5	3.43	25	29.9%	-7.95 [-10.21, -5.69]
Yuan J. 2019	5	1.75	20	6	2	20	34.6%	-1.00 [-2.16, 0.16]
**Total (95% CI)**			**87**			**87**	**100.0%**	**-3.37 [-6.24, -0.49]**
Heterogeneity: Tau² = 5.86; Chi² = 29.70, df = 2 (P < 0.00001); I² = 93%								
Test for overall effect: Z = 2.30 (P = 0.02)								

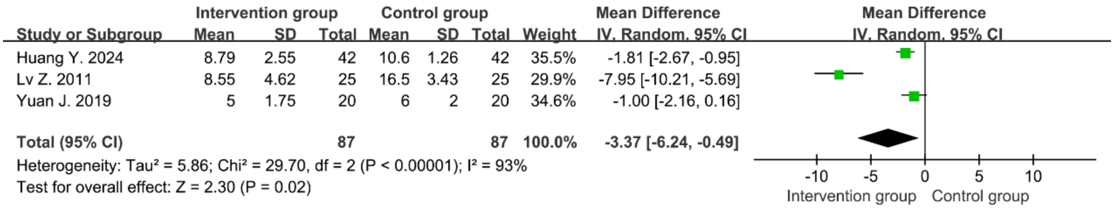

Supplementary Figure S4. Subgroup analysis of $CD4^+/CD8^+$ ratio based on language

	Intervention group			Control group				Mean Difference
Study or Subgroup	Mean	SD	Total	Mean	SD	Total	Weight	IV, Fixed, 95% CI
**1.4.1 English**								
Wang X. 2011	1.72	0.55	12	1.35	0.65	12	12.4%	0.37 [-0.11, 0.85]
**Subtotal (95% CI)**			**12**			**12**	**12.4%**	**0.37 [-0.11, 0.85]**
Heterogeneity: Not applicable								
Test for overall effect: Z = 1.51 (P = 0.13)								
**1.4.2 Chinese**								
Huang Y. 2024	2.95	0.36	42	1.99	0.58	42	67.5%	0.96 [0.75, 1.17]
Yuan J. 2019	1.88	0.71	20	1.37	0.49	20	20.1%	0.51 [0.13, 0.89]
**Subtotal (95% CI)**			**62**			**62**	**87.6%**	**0.86 [0.68, 1.04]**
Heterogeneity: Chi² = 4.19, df = 1 (P = 0.04); I² = 76%								
Test for overall effect: Z = 9.27 (P < 0.00001)								
**Total (95% CI)**			**74**			**74**	**100.0%**	**0.80 [0.63, 0.97]**
Heterogeneity: Chi² = 7.63, df = 2 (P = 0.02); I² = 74%								
Test for overall effect: Z = 9.20 (P < 0.00001)								
Test for subgroup differences: Chi² = 3.43, df = 1 (P = 0.06), I² = 70.9%								

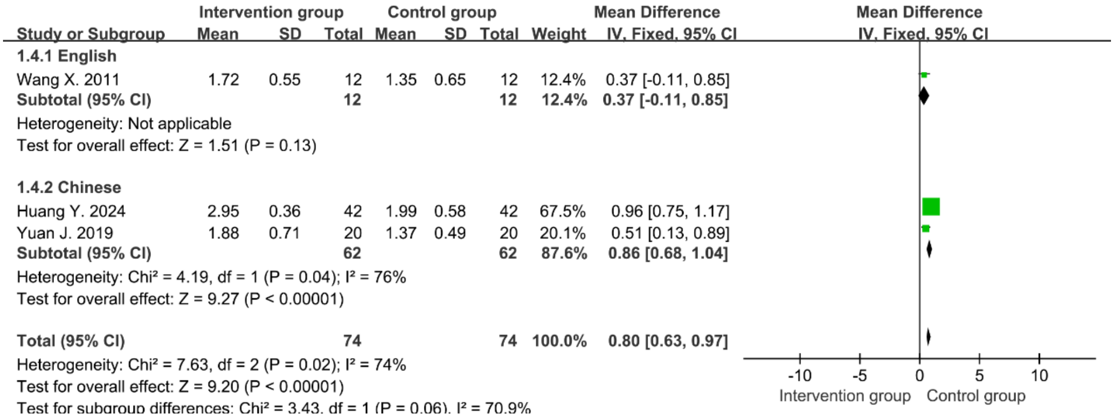

Supplementary Figure S5. Subgroup analysis of C-reactive protein levels based on language

	Intervention group			Control group				Mean Difference
Study or Subgroup	Mean	SD	Total	Mean	SD	Total	Weight	IV, Random, 95% CI
**1.5.1 English**								
Ke L. 2022	53.6	54.2	254	54.5	54.3	254	24.0%	-0.90 [-10.34, 8.54]
**Subtotal (95% CI)**			**254**			**254**	**24.0%**	**-0.90 [-10.34, 8.54]**
Heterogeneity: Not applicable								
Test for overall effect: Z = 0.19 (P = 0.85)								
**1.5.2 Chinese**								
Huang Y. 2024	18.83	1.67	42	45.21	4.13	42	27.7%	-26.38 [-27.73, -25.03]
Lv Z. 2011	104.4	20.16	25	140.34	21.25	25	22.5%	-35.94 [-47.42, -24.46]
Yuan J. 2019	31.62	8.23	20	43.1	12.1	20	25.9%	-11.48 [-17.89, -5.07]
**Subtotal (95% CI)**			**87**			**87**	**76.0%**	**-23.93 [-35.38, -12.48]**
Heterogeneity: Tau² = 89.05; Chi² = 22.84, df = 2 (P < 0.0001); I² = 91%								
Test for overall effect: Z = 4.10 (P < 0.0001)								
**Total (95% CI)**			**341**			**341**	**100.0%**	**-18.57 [-31.09, -6.04]**
Heterogeneity: Tau² = 147.20; Chi² = 49.27, df = 3 (P < 0.00001); I² = 94%								
Test for overall effect: Z = 2.91 (P = 0.004)								
Test for subgroup differences: Chi² = 9.25, df = 1 (P = 0.002), I² = 89.2%								

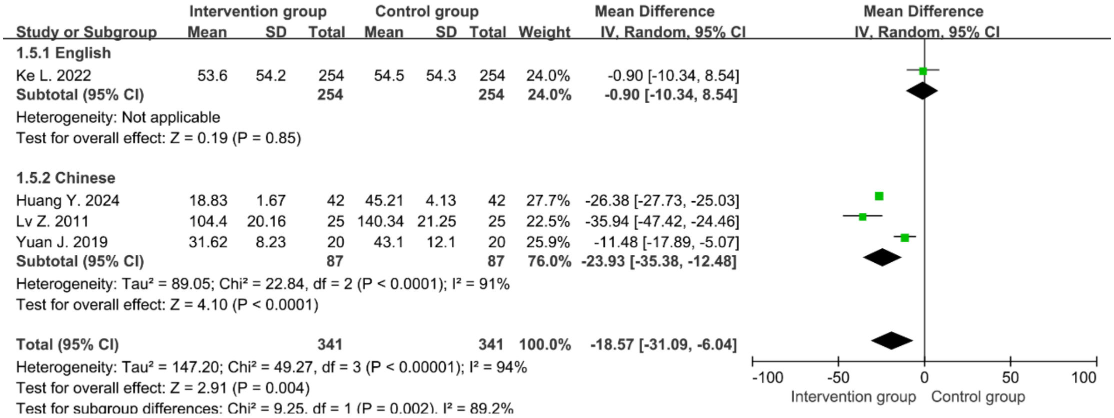

Supplementary Figure S6. Subgroup analysis of positive blood culture based on language

	Intervention group		Control group			Risk Ratio
Study or Subgroup	Events	Total	Events	Total	Weight	M-H, Fixed, 95% CI
**1.6.1 English**						
Ke L. 2022	18	254	25	254	59.5%	0.72 [0.40, 1.29]
Wang X. 2011	2	12	9	12	21.4%	0.22 [0.06, 0.82]
**Subtotal (95% CI)**		**266**		**266**	**81.0%**	**0.59 [0.35, 0.99]**
Total events	20		34			
Heterogeneity: Chi² = 2.60, df = 1 (P = 0.11); I² = 62%						
Test for overall effect: Z = 2.01 (P = 0.04)						
**1.6.2 Chinese**						
Yuan J. 2019	5	20	8	20	19.0%	0.63 [0.25, 1.58]
**Subtotal (95% CI)**		**20**		**20**	**19.0%**	**0.63 [0.25, 1.58]**
Total events	5		8			
Heterogeneity: Not applicable						
Test for overall effect: Z = 0.99 (P = 0.32)						
**Total (95% CI)**		**286**		**286**	**100.0%**	**0.60 [0.38, 0.94]**
Total events	25		42			
Heterogeneity: Chi² = 2.61, df = 2 (P = 0.27); I² = 23%						
Test for overall effect: Z = 2.24 (P = 0.03)						
Test for subgroup differences: Chi² = 0.01, df = 1 (P = 0.91), I² = 0%						

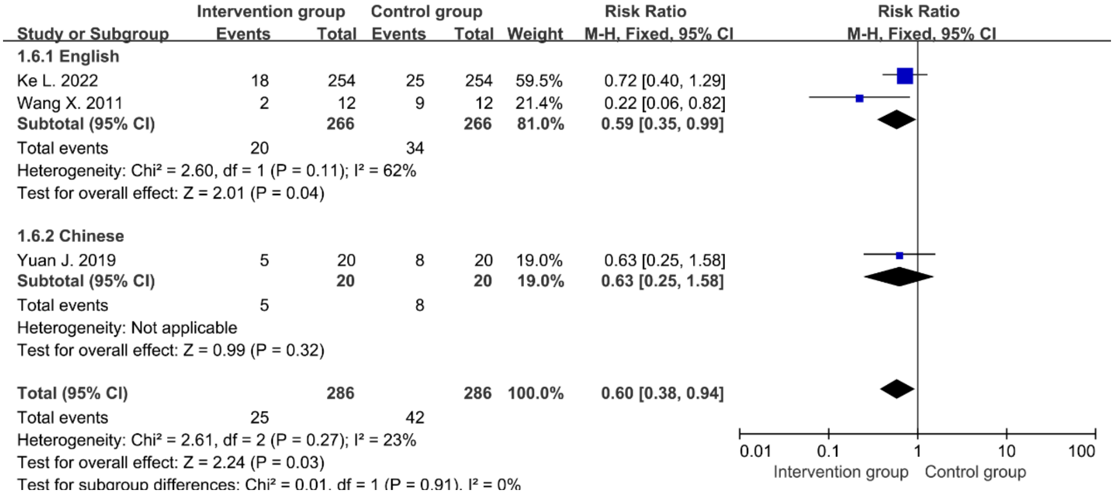

Supplementary Table S2. Risk assessment of bias for Ke L. et al.'s study using Cochrane RoB 2.0

**Cochrane RoB 2.0 Checklist**			
Version 2 of the Cochrane risk-of-bias assessment tool for randomised trials: bias domains, signalling questions, response options, and risk-of-bias judgments			
Bias domain and signalling question*	Response options		
	Lower risk of bias	Higher risk of bias	Other
**1. Bias arising from the randomisation process**			
1.1 Was the allocation sequence random?	Y/PY	N/PN	NI
	Y		
1.2 Was the allocation sequence concealed until participants were enrolled and assigned to interventions?	Y/PY	N/PN	NI
	Y		
1.3 Did baseline differences between intervention groups suggest a problem with the randomisation process?	N/PN	Y/PY	NI
	N		
Risk-of-bias judgment (low/high/some concerns)			
	low		
Optional: What is the predicted direction of bias arising from the randomisation process?			
**2. Bias due to deviations from intended interventions**			
2.1 Were participants aware of their assigned intervention during the trial?	N/PN	Y/PY	NI
	N		
2.2 Were carers and people delivering the interventions aware of participants' assigned intervention during the trial?	N/PN	Y/PY	NI
	N		
2.3 If Y/PY/NI to 2.1 or 2.2: Were there deviations from the intended intervention that arose because of the trial context?	N/PN	Y/PY	NA/NI
2.4 If Y/PY/NI to 2.3: Were these deviations likely to have affected the outcome?	N/PN	Y/PY	NA/NI
2.5 If Y/PY to 2.4: Were these deviations from intended intervention balanced between groups?	Y/PY	N/PN	NA/NI

2.6 Was an appropriate analysis used to estimate the effect of assignment to intervention?	Y/PY	N/PN	NI
	Y		
2.7 If N/PN/NI to 2.6: Was there potential for a substantial impact (on the result) of the failure to analyse participants in the group to which they were randomised?	N/PN	Y/PY	NA/NI
Risk-of-bias judgment (low/high/some concerns)			
	low		
Optional: What is the predicted direction of bias due to deviations from intended interventions?			
**3. Bias due to missing outcome data**			
3.1 Were data for this outcome available for all, or nearly all, participants randomised?	Y/PY	N/PN	NI
	Y		
3.2 If N/PN/NI to 3.1: Is there evidence that the result was not biased by missing outcome data?	Y/PY	N/PN	NA
3.3 If N/PN to 3.2: Could missingness in the outcome depend on its true value?	N/PN	Y/PY	NA/NI
3.4 If Y/PY/NI to 3.3: Is it likely that missingness in the outcome depended on its true value?	N/PN	Y/PY	NA/NI
Risk-of-bias judgment (low/high/some concerns)			
	low		
Optional: What is the predicted direction of bias due to missing outcome data?			
**4. Bias in measurement of the outcome**			
4.1 Was the method of measuring the outcome inappropriate?	N/PN	Y/PY	NI
	N		
4.2 Could measurement or ascertainment of the outcome have differed between intervention groups?	N/PN	Y/PY	NI
	N		
4.3 If N/PN/NI to 4.1 and 4.2: Were outcome assessors aware of the intervention received by study participants?	N/PN	Y/PY	NI

4.4 If Y/PY/NI to 4.3: Could assessment of the outcome have been influenced by knowledge of intervention received?	N/PN	Y/PY	NA/NI
4.5 If Y/PY/NI to 4.4: Is it likely that assessment of the outcome was influenced by knowledge of intervention received?	N/PN	Y/PY	NA/NI
Risk-of-bias judgment (low/high/some concerns)			
	low		
Optional: What is the predicted direction of bias in measurement of the outcome?			
**5. Bias in selection of the reported result**			
5.1 Were the data that produced this result analysed in accordance with a prespecified analysis plan that was finalised before unblinded outcome data were available for analysis?	Y/PY	N/PN	NI
	Y		
**Is the numerical result being assessed likely to have been selected, on the basis of the results, from:**			
5.2 Multiple eligible outcome measurements (eg, scales, definitions, time points) within the outcome domain?	N/PN	Y/PY	NI
	N		
5.3 Multiple eligible analyses of the data?	N/PN	Y/PY	NI
	N		
Risk-of-bias judgment (low/high/some concerns)			
	low		
Optional: What is the predicted direction bias due to selection of the reported results?			
**Overall bias**			
Risk-of-bias judgment (low/high/some concerns)			
	low		
Optional: What is the overall predicted direction of bias for this outcome?			
Y=yes; PY=probably yes; PN=probably no; N=no; NA=not applicable; NI=no information.			
*Signalling questions for bias due to deviations from intended interventions relate to the effect of assignment to intervention.			
	N		

Supplementary Table S3. Risk assessment of bias for Wang X. et al.'s study using Cochrane RoB 2.0

Cochrane RoB 2.0 Checklist			
Version 2 of the Cochrane risk-of-bias assessment tool for randomised trials: bias domains, signalling questions, response options, and risk-of-bias judgments			
Bias domain and signalling question*	Response options		
	Lower risk of bias	Higher risk of bias	Other
**1. Bias arising from the randomisation process**			
1.1 Was the allocation sequence random?	Y/PY	N/PN	NI
	Y		
1.2 Was the allocation sequence concealed until participants were enrolled and assigned to interventions?	Y/PY	N/PN	NI
	PY		
1.3 Did baseline differences between intervention groups suggest a problem with the randomisation process?	N/PN	Y/PY	NI
	N		
Risk-of-bias judgment (low/high/some concerns)			
	low		
Optional: What is the predicted direction of bias arising from the randomisation process?			
**2. Bias due to deviations from intended interventions**			
2.1 Were participants aware of their assigned intervention during the trial?	N/PN	Y/PY	NI
			NI
2.2 Were carers and people delivering the interventions aware of participants' assigned intervention during the trial?	N/PN	Y/PY	NI
	N		
2.3 If Y/PY/NI to 2.1 or 2.2: Were there deviations from the intended intervention that arose because of the trial context?	N/PN	Y/PY	NA/NI
	N		
2.4 If Y/PY/NI to 2.3: Were these deviations likely to have affected the outcome?	N/PN	Y/PY	NA/NI
2.5 If Y/PY to 2.4: Were these deviations from intended intervention balanced between groups?	Y/PY	N/PN	NA/NI

2.6 Was an appropriate analysis used to estimate the effect of assignment to intervention?	Y/PY	N/PN	NI
	Y		
2.7 If N/PN/NI to 2.6: Was there potential for a substantial impact (on the result) of the failure to analyse participants in the group to which they were randomised?	N/PN	Y/PY	NA/NI
Risk-of-bias judgment (low/high/some concerns)			
	low		
Optional: What is the predicted direction of bias due to deviations from intended interventions?			
**3. Bias due to missing outcome data**			
3.1 Were data for this outcome available for all, or nearly all, participants randomised?	Y/PY	N/PN	NI
	Y		
3.2 If N/PN/NI to 3.1: Is there evidence that the result was not biased by missing outcome data?	Y/PY	N/PN	NA
3.3 If N/PN to 3.2: Could missingness in the outcome depend on its true value?	N/PN	Y/PY	NA/NI
3.4 If Y/PY/NI to 3.3: Is it likely that missingness in the outcome depended on its true value?	N/PN	Y/PY	NA/NI
Risk-of-bias judgment (low/high/some concerns)			
	low		
Optional: What is the predicted direction of bias due to missing outcome data?			
**4. Bias in measurement of the outcome**			
4.1 Was the method of measuring the outcome inappropriate?	N/PN	Y/PY	NI
	N		
4.2 Could measurement or ascertainment of the outcome have differed between intervention groups?	N/PN	Y/PY	NI
	N		
4.3 If N/PN/NI to 4.1 and 4.2: Were outcome assessors aware of the intervention received by study participants?	N/PN	Y/PY	NI

4.4 If Y/PY/NI to 4.3: Could assessment of the outcome have been influenced by knowledge of intervention received?	N/PN	Y/PY	NA/NI
4.5 If Y/PY/NI to 4.4: Is it likely that assessment of the outcome was influenced by knowledge of intervention received?	N/PN	Y/PY	NA/NI
Risk-of-bias judgment (low/high/some concerns)			
	low		
Optional: What is the predicted direction of bias in measurement of the outcome?			
**5. Bias in selection of the reported result**			
5.1 Were the data that produced this result analysed in accordance with a prespecified analysis plan that was finalised before unblinded outcome data were available for analysis?	Y/PY	N/PN	NI
	Y		
**Is the numerical result being assessed likely to have been selected, on the basis of the results, from:**			
5.2 Multiple eligible outcome measurements (eg, scales, definitions, time points) within the outcome domain?	N/PN	Y/PY	NI
	N		
5.3 Multiple eligible analyses of the data?	N/PN	Y/PY	NI
	N		
Risk-of-bias judgment (low/high/some concerns)			
	low		
Optional: What is the predicted direction bias due to selection of the reported results?			
**Overall bias**			
Risk-of-bias judgment (low/high/some concerns)			
	low		
Optional: What is the overall predicted direction of bias for this outcome?			
Y=yes; PY=probably yes; PN=probably no; N=no; NA=not applicable; NI=no information.			
*Signalling questions for bias due to deviations from intended interventions relate to the effect of assignment to intervention.			
	N		

Supplementary Table S4. Risk assessment of bias for Yuan J. et al.'s study using Cochrane RoB 2.0

**Cochrane RoB 2.0 Checklist**			
Version 2 of the Cochrane risk-of-bias assessment tool for randomised trials: bias domains, signalling questions, response options, and risk-of-bias judgments			
Bias domain and signalling question*	Response options		
	Lower risk of bias	Higher risk of bias	Other
**1. Bias arising from the randomisation process**			
1.1 Was the allocation sequence random?	Y/PY	N/PN	NI
	Y		
1.2 Was the allocation sequence concealed until participants were enrolled and assigned to interventions?	Y/PY	N/PN	NI
			NI
1.3 Did baseline differences between intervention groups suggest a problem with the randomisation process?	N/PN	Y/PY	NI
	N		
Risk-of-bias judgment (low/high/some concerns)			
	low		
Optional: What is the predicted direction of bias arising from the randomisation process?			
**2. Bias due to deviations from intended interventions**			
2.1 Were participants aware of their assigned intervention during the trial?	N/PN	Y/PY	NI
			NI
2.2 Were carers and people delivering the interventions aware of participants' assigned intervention during the trial?	N/PN	Y/PY	NI
			NI
2.3 If Y/PY/NI to 2.1 or 2.2: Were there deviations from the intended intervention that arose because of the trial context?	N/PN	Y/PY	NA/NI
	N		
2.4 If Y/PY/NI to 2.3: Were these deviations likely to have affected the outcome?	N/PN	Y/PY	NA/NI
2.5 If Y/PY to 2.4: Were these deviations from intended intervention balanced between groups?	Y/PY	N/PN	NA/NI

2.6 Was an appropriate analysis used to estimate the effect of assignment to intervention?	Y/PY	N/PN	NI
	Y		
2.7 If N/PN/NI to 2.6: Was there potential for a substantial impact (on the result) of the failure to analyse participants in the group to which they were randomised?	N/PN	Y/PY	NA/NI
Risk-of-bias judgment (low/high/some concerns)			
			some concerns
Optional: What is the predicted direction of bias due to deviations from intended interventions?			
**3. Bias due to missing outcome data**			
3.1 Were data for this outcome available for all, or nearly all, participants randomised?	Y/PY	N/PN	NI
	Y		
3.2 If N/PN/NI to 3.1: Is there evidence that the result was not biased by missing outcome data?	Y/PY	N/PN	NA
3.3 If N/PN to 3.2: Could missingness in the outcome depend on its true value?	N/PN	Y/PY	NA/NI
3.4 If Y/PY/NI to 3.3: Is it likely that missingness in the outcome depended on its true value?	N/PN	Y/PY	NA/NI
Risk-of-bias judgment (low/high/some concerns)			
	low		
Optional: What is the predicted direction of bias due to missing outcome data?			
**4. Bias in measurement of the outcome**			
4.1 Was the method of measuring the outcome inappropriate?	N/PN	Y/PY	NI
	N		
4.2 Could measurement or ascertainment of the outcome have differed between intervention groups?	N/PN	Y/PY	NI
	N		
4.3 If N/PN/NI to 4.1 and 4.2: Were outcome assessors aware of the intervention received by study participants?	N/PN	Y/PY	NI

4.4 If Y/PY/NI to 4.3: Could assessment of the outcome have been influenced by knowledge of intervention received?	N/PN	Y/PY	NA/NI
4.5 If Y/PY/NI to 4.4: Is it likely that assessment of the outcome was influenced by knowledge of intervention received?	N/PN	Y/PY	NA/NI
Risk-of-bias judgment (low/high/some concerns)			
	low		
Optional: What is the predicted direction of bias in measurement of the outcome?			
**5. Bias in selection of the reported result**			
5.1 Were the data that produced this result analysed in accordance with a prespecified analysis plan that was finalised before unblinded outcome data were available for analysis?	Y/PY	N/PN	NI
	Y		
**Is the numerical result being assessed likely to have been selected, on the basis of the results, from:**			
5.2 Multiple eligible outcome measurements (eg, scales, definitions, time points) within the outcome domain?	N/PN	Y/PY	NI
	N		
5.3 Multiple eligible analyses of the data?	N/PN	Y/PY	NI
	N		
Risk-of-bias judgment (low/high/some concerns)			
	low		
Optional: What is the predicted direction bias due to selection of the reported results?			
**Overall bias**			
Risk-of-bias judgment (low/high/some concerns)			
			some concerns
Optional: What is the overall predicted direction of bias for this outcome?			
Y=yes; PY=probably yes; PN=probably no; N=no; NA=not applicable; NI=no information.			
*Signalling questions for bias due to deviations from intended interventions relate to the effect of assignment to intervention.			
	N		

Supplementary Table S5. Risk assessment of bias for Lv Z. et al.'s study using Cochrane RoB 2.0

**Cochrane RoB 2.0 Checklist**			
Version 2 of the Cochrane risk-of-bias assessment tool for randomised trials: bias domains, signalling questions, response options, and risk-of-bias judgments			
Bias domain and signalling question*	Response options		
	Lower risk of bias	Higher risk of bias	Other
**1. Bias arising from the randomisation process**			
1.1 Was the allocation sequence random?	Y/PY	N/PN	NI
	Y		
1.2 Was the allocation sequence concealed until participants were enrolled and assigned to interventions?	Y/PY	N/PN	NI
	PY		
1.3 Did baseline differences between intervention groups suggest a problem with the randomisation process?	N/PN	Y/PY	NI
	N		
Risk-of-bias judgment (low/high/some concerns)			
	low		
Optional: What is the predicted direction of bias arising from the randomisation process?			
**2. Bias due to deviations from intended interventions**			
2.1 Were participants aware of their assigned intervention during the trial?	N/PN	Y/PY	NI
			NI
2.2 Were carers and people delivering the interventions aware of participants' assigned intervention during the trial?	N/PN	Y/PY	NI
			NI
2.3 If Y/PY/NI to 2.1 or 2.2: Were there deviations from the intended intervention that arose because of the trial context?	N/PN	Y/PY	NA/NI
	N		
2.4 If Y/PY/NI to 2.3: Were these deviations likely to have affected the outcome?	N/PN	Y/PY	NA/NI
2.5 If Y/PY to 2.4: Were these deviations from intended intervention balanced between groups?	Y/PY	N/PN	NA/NI

2.6 Was an appropriate analysis used to estimate the effect of assignment to intervention?	Y/PY	N/PN	NI
	Y		
2.7 If N/PN/NI to 2.6: Was there potential for a substantial impact (on the result) of the failure to analyse participants in the group to which they were randomised?	N/PN	Y/PY	NA/NI
Risk-of-bias judgment (low/high/some concerns)			
			some concerns
Optional: What is the predicted direction of bias due to deviations from intended interventions?			
**3. Bias due to missing outcome data**			
3.1 Were data for this outcome available for all, or nearly all, participants randomised?	Y/PY	N/PN	NI
	PY		
3.2 If N/PN/NI to 3.1: Is there evidence that the result was not biased by missing outcome data?	Y/PY	N/PN	NA
3.3 If N/PN to 3.2: Could missingness in the outcome depend on its true value?	N/PN	Y/PY	NA/NI
3.4 If Y/PY/NI to 3.3: Is it likely that missingness in the outcome depended on its true value?	N/PN	Y/PY	NA/NI
Risk-of-bias judgment (low/high/some concerns)			
	low		
Optional: What is the predicted direction of bias due to missing outcome data?			
**4. Bias in measurement of the outcome**			
4.1 Was the method of measuring the outcome inappropriate?	N/PN	Y/PY	NI
	N		
4.2 Could measurement or ascertainment of the outcome have differed between intervention groups?	N/PN	Y/PY	NI
	N		
4.3 If N/PN/NI to 4.1 and 4.2: Were outcome assessors aware of the intervention received by study participants?	N/PN	Y/PY	NI

4.4 If Y/PY/NI to 4.3: Could assessment of the outcome have been influenced by knowledge of intervention received?	N/PN	Y/PY	NA/NI
4.5 If Y/PY/NI to 4.4: Is it likely that assessment of the outcome was influenced by knowledge of intervention received?	N/PN	Y/PY	NA/NI
Risk-of-bias judgment (low/high/some concerns)			
	low		
Optional: What is the predicted direction of bias in measurement of the outcome?			
**5. Bias in selection of the reported result**			
5.1 Were the data that produced this result analysed in accordance with a prespecified analysis plan that was finalised before unblinded outcome data were available for analysis?	Y/PY	N/PN	NI
	Y		
**Is the numerical result being assessed likely to have been selected, on the basis of the results, from:**			
5.2 Multiple eligible outcome measurements (eg, scales, definitions, time points) within the outcome domain?	N/PN	Y/PY	NI
			NI
5.3 Multiple eligible analyses of the data?	N/PN	Y/PY	NI
			NI
Risk-of-bias judgment (low/high/some concerns)			
			some concerns
Optional: What is the predicted direction bias due to selection of the reported results?			
**Overall bias**			
Risk-of-bias judgment (low/high/some concerns)			
			some concerns
Optional: What is the overall predicted direction of bias for this outcome?			
Y=yes; PY=probably yes; PN=probably no; N=no; NA=not applicable; NI=no information.			
*Signalling questions for bias due to deviations from intended interventions relate to the effect of assignment to intervention.			
	N		

Supplementary Table S6. Risk assessment of bias for Huang Y. et al.'s study using Cochrane RoB 2.0

**Cochrane RoB 2.0 Checklist**			
Version 2 of the Cochrane risk-of-bias assessment tool for randomised trials: bias domains, signalling questions, response options, and risk-of-bias judgments			
Bias domain and signalling question*	Response options		
	Lower risk of bias	Higher risk of bias	Other
**1. Bias arising from the randomisation process**			
1.1 Was the allocation sequence random?	Y/PY	N/PN	NI
	Y		
1.2 Was the allocation sequence concealed until participants were enrolled and assigned to interventions?	Y/PY	N/PN	NI
			NI
1.3 Did baseline differences between intervention groups suggest a problem with the randomisation process?	N/PN	Y/PY	NI
	N		
Risk-of-bias judgment (low/high/some concerns)			
	low		
Optional: What is the predicted direction of bias arising from the randomisation process?			
**2. Bias due to deviations from intended interventions**			
2.1 Were participants aware of their assigned intervention during the trial?	N/PN	Y/PY	NI
			NI
2.2 Were carers and people delivering the interventions aware of participants' assigned intervention during the trial?	N/PN	Y/PY	NI
			NI
2.3 If Y/PY/NI to 2.1 or 2.2: Were there deviations from the intended intervention that arose because of the trial context?	N/PN	Y/PY	NA/NI
	N		
2.4 If Y/PY/NI to 2.3: Were these deviations likely to have affected the outcome?	N/PN	Y/PY	NA/NI
2.5 If Y/PY to 2.4: Were these deviations from intended intervention balanced between groups?	Y/PY	N/PN	NA/NI

2.6 Was an appropriate analysis used to estimate the effect of assignment to intervention?	Y/PY	N/PN	NI
	Y		
2.7 If N/PN/NI to 2.6: Was there potential for a substantial impact (on the result) of the failure to analyse participants in the group to which they were randomised?	N/PN	Y/PY	NA/NI
Risk-of-bias judgment (low/high/some concerns)			
			some concerns
Optional: What is the predicted direction of bias due to deviations from intended interventions?			
**3. Bias due to missing outcome data**			
3.1 Were data for this outcome available for all, or nearly all, participants randomised?	Y/PY	N/PN	NI
	PY		
3.2 If N/PN/NI to 3.1: Is there evidence that the result was not biased by missing outcome data?	Y/PY	N/PN	NA
3.3 If N/PN to 3.2: Could missingness in the outcome depend on its true value?	N/PN	Y/PY	NA/NI
3.4 If Y/PY/NI to 3.3: Is it likely that missingness in the outcome depended on its true value?	N/PN	Y/PY	NA/NI
Risk-of-bias judgment (low/high/some concerns)			
	low		
Optional: What is the predicted direction of bias due to missing outcome data?			
**4. Bias in measurement of the outcome**			
4.1 Was the method of measuring the outcome inappropriate?	N/PN	Y/PY	NI
	N		
4.2 Could measurement or ascertainment of the outcome have differed between intervention groups?	N/PN	Y/PY	NI
	N		
4.3 If N/PN/NI to 4.1 and 4.2: Were outcome assessors aware of the intervention received by study participants?	N/PN	Y/PY	NI

4.4 If Y/PY/NI to 4.3: Could assessment of the outcome have been influenced by knowledge of intervention received?	N/PN	Y/PY	NA/NI
4.5 If Y/PY/NI to 4.4: Is it likely that assessment of the outcome was influenced by knowledge of intervention received?	N/PN	Y/PY	NA/NI
Risk-of-bias judgment (low/high/some concerns)			
	low		
Optional: What is the predicted direction of bias in measurement of the outcome?			
**5. Bias in selection of the reported result**			
5.1 Were the data that produced this result analysed in accordance with a prespecified analysis plan that was finalised before unblinded outcome data were available for analysis?	Y/PY	N/PN	NI
	Y		
**Is the numerical result being assessed likely to have been selected, on the basis of the results, from:**			
5.2 Multiple eligible outcome measurements (eg, scales, definitions, time points) within the outcome domain?	N/PN	Y/PY	NI
	N		
5.3 Multiple eligible analyses of the data?	N/PN	Y/PY	NI
	N		
Risk-of-bias judgment (low/high/some concerns)			
	low		
Optional: What is the predicted direction bias due to selection of the reported results?			
**Overall bias**			
Risk-of-bias judgment (low/high/some concerns)			
			some concerns
Optional: What is the overall predicted direction of bias for this outcome?			
Y=yes; PY=probably yes; PN=probably no; N=no; NA=not applicable; NI=no information.			
*Signalling questions for bias due to deviations from intended interventions relate to the effect of assignment to intervention.			
	N		

Supplementary Table S7. Overall bias for all studies using Cochrane RoB 2.0

Study	Randomisation Process	Deviations From Intended Interventions	Missing Outcome Data	Measurement of Outcome	Selection of Reported Result	Result Evaluation	Overall Bias
Ke L. et al[30]	Low	Low	Low	Low	Low	Low	Low
Wang X. et al[31]	Low	Low	Low	Low	Low	Low	Low
Yuan J. et al[32]	Low	Some concerns	Low	Low	Low	Low	Some concerns
Lv Z. et al[33]	Low	Some concerns	Low	Low	Low	Some concerns	Some concerns
Huang Y. et al[34]	Low	Some concerns	Low	Low	Low	Low	Some concerns

Supplementary Figure S7. Diagram for main subsets and partial functions of $CD4^{+}$ T cells differentiation

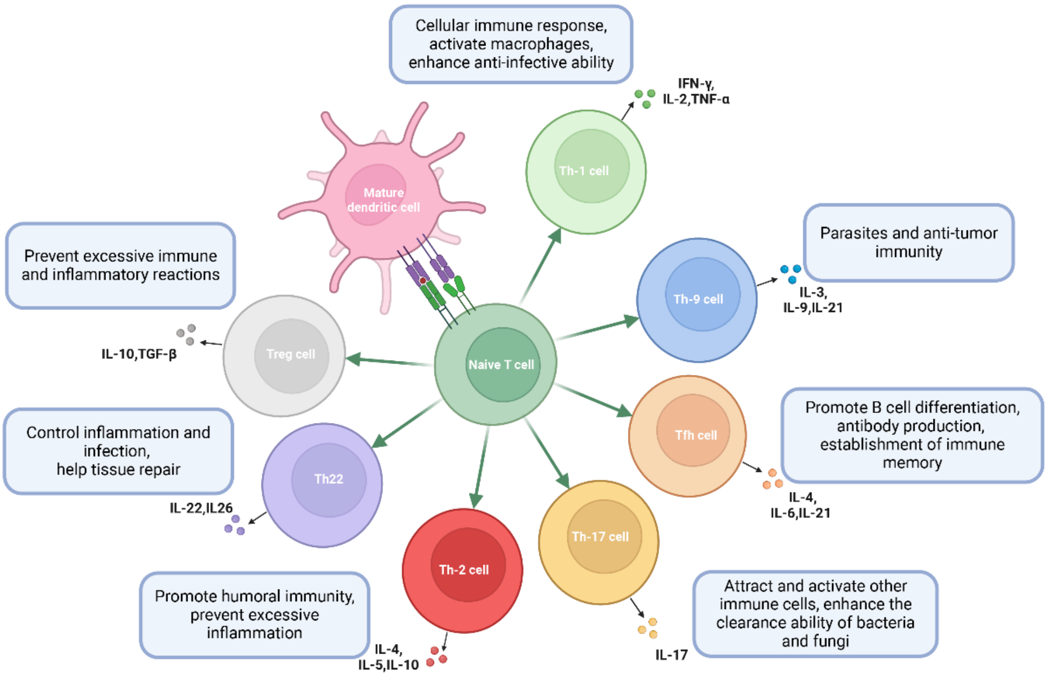

Supplement: Supplementary file 1 [file DataSheet1.pdf]
